# Targeting Bacterial Biofilms by the Green Tea Polyphenol EGCG

**DOI:** 10.3390/molecules24132403

**Published:** 2019-06-29

**Authors:** Regine Hengge

**Affiliations:** Institut für Biologie/Mikrobiologie, Humboldt-Universität zu Berlin, 10155 Berlin, Germany; regine.hengge@hu-berlin.de; Tel.: +49-30-2093-49686

**Keywords:** bacterial biofilm, functional amyloid, curli fibre, bacterial exopolysaccharides, bacterial cellulose, chronic infection, antimicrobial

## Abstract

Bacterial biofilms are multicellular aggregates in which cells are embedded in an extracellular matrix of self-produced biopolymers. Being refractory to antibiotic treatment and host immune systems, biofilms are involved in most chronic infections, and anti-biofilm agents are being searched for urgently. Epigallocatechin-3-gallate (EGCG) was recently shown to act against biofilms by strongly interfering with the assembly of amyloid fibres and the production of phosphoethanolamin-modified cellulose fibrils. Mechanistically, this includes a direct inhibition of the fibre assembly, but also triggers a cell envelope stress response that down-regulates the synthesis of these widely occurring biofilm matrix polymers. Based on its anti-amyloidogenic properties, EGCG seems useful against biofilms involved in cariogenesis or chronic wound infection. However, EGCG seems inefficient against or may even sometimes promote biofilms which rely on other types of matrix polymers, suggesting that searching for ‘magic bullet’ anti-biofilm agents is an unrealistic goal. Combining molecular and ecophysiological aspects in this review also illustrates why plants control the formation of biofilms on their surfaces by producing anti-amyloidogenic compounds such as EGCG. These agents are not only helpful in combating certain biofilms in chronic infections but even seem effective against the toxic amyloids associated with neuropathological diseases.

## 1. Introduction

Bacterial biofilms are ubiquitous multicellular aggregates which are usually attached to biotic or abiotic surfaces in which bacteria are embedded in an extracellular matrix of self-produced polymers [1,2,3,4]. Bacteria in biofilms are refractory to antibiotic treatment and host immune systems [5,6]. As a consequence, biofilms of pathogenic bacteria play crucial roles in chronic infections (e.g., of wounds, soft tissue and the urinary tract or in cystic fibrosis (CF), laryngitis, otitis media, dental plaque, caries and periodontosis) as well as in the colonization of catheters, artificial heart valves, orthopedic implants and other intracorporal medical devices [7,8]. Notably, this biofilm-associated antibiotic tolerance is not acquired by horizontal gene transfer or specific mutations in antibiotic targets as in classical antibiotic resistance, but it is part of the normal bacterial physiological and regulatory repertoire.

With biofilms being key to chronic infections, the need for anti-biofilm compounds has become apparent [8,9,10,11]. Unlike classical antibiotics, these are active at concentrations that do not interfere with bacterial growth or survival but inhibit the formation of bacterial biofilms or disrupt already existing biofilms by targeting biofilm-related structures or regulation. Thereby, they can act synergistically with antibiotics and/or allow the immune system to attack bacteria that would otherwise be hidden away in biofilms [9,10,11,12,13]. Screening large chemical libraries using high-throughput approaches has been used to identify anti-biofilm compounds [14]. Alternatively, natural products originating from plants, marine sponges or other biological sources have been tested [9,15,16,17]. The latter approach has revealed that numerous compounds, which naturally occur in plants, as well as complex plant extracts indeed exhibit anti-biofilm activity [18].

Epigallocatechin-3-gallate (EGCG) and related catechins, which are not only the major polyphenols in green tea (*Camellia sinensis*) but are also present in many other plants, have long been known to have numerous benefits for human health (see other articles in this compendium; for a review, see [19]). This also led to the first studies of EGCG’s effects on bacteria almost 20 years ago. After a short introduction to biofims, this review will summarize reports on antimicrobial and above all anti-biofilm activities of EGCG with a focus on the more recent studies that have addressed the molecular mechanisms of action of EGCG. In addition, some bacterio-ecophysiological considerations will suggest that plants may be a highly valuable source of many more anti-biofilm agents for which EGCG may serve as a paradigm for the identification and analyses of mechanisms of action.

## 2. Bacterial Biofilms

The hallmark of bacterial biofilms is the presence of an extracellular polymeric matrix of complex and variable composition [20,21]. This matrix often literally encases the bacterial cells and confers structural stability, cohesion and elasticity to the biofilm, i.e., tissue-like properties, as most evident from the buckling, folding and wrinkling of colony biofilms that form large-scale three-dimensional morphological patterns [22,23,24,25]. Importantly, the matrix confers protection—for example, by trapping phages or antimicrobial compounds and preventing access of predators—and by binding water, it contributes to homeostatic conditions within the biofilm [3,6,21,26].

Biofilm matrices contain secreted proteins, some of which can form amyloid fibres, exopolysaccharides and extracellular DNA, collectively termed extracellular polymeric substances (EPS) [21]. Exopolysaccharides, which differ in their monosaccharide constituents, chemical modifications, composition and types of glycosidic linkage, can be divided into two functional classes with either aggregative or water-binding mucoid properties. A widely occurring example of aggregative exopolysaccharides, which in general confer cohesion and structural stability to biofilms, is cellulose [27,28,29]. In biofilm matrices of *Escherichia coli* and many other bacteria, cellulose is present as a phosphoethanolamin-modified derivative (pEtN-cellulose) [30]. Other aggregative exopolysaccharides are Psl and Pel of *Pseudomonas* [31], VPS of *Vibrio cholerae* [32] or the exopolysaccharide of *Bacillus subtilis* [20]. Water-binding mucoid exopolysaccharides include colanic acid in enteric bacteria [33], alginate in *Pseudomonas aeruginosa* [34] or other capsule polysaccharides. Exopolysaccharides are synthesized by inner membrane-associated glycosyltransferases, which form the core of larger synthesis and secretion complexes that include additional chaperone and pore components in the cell envelope that guide the nascent polysaccharides to the cell surface [35]. Also, the pEtN group is transferred to cellulose during its transit through the periplasm of *E. coli* and other Gram-negative bacteria [30].

Amyloid fibres are found in most natural biofilms [36,37,38,39]. Although differing in the sequences of their protein subunits, amyloid fibres exhibit similar molecular superstructures classically consisting of cross-beta sheets that further assemble into fibrils and finally fibres [39]. These are insoluble in sodium dodecyl sulfate (SDS), proteinase K resistant and typically stain with Congo red (CR) and thioflavin T/S. Recent data indicate a certain structural variability that also includes cross-beta helical structures in some of these fibres, with cross-beta sheet conformations being promoted by low pH. Seeds of these structures are able to template additional subunits into amyloid fibres (recently summarized in [39]). From the perspective of bacteria, these fibres are ‘functional’ amyloids as they contribute to aggregation and protective properties of biofilms [39] and can also serve the bacteria as virulence factors in a host environment [40]. This is in contrast to the toxic amyloid fibres and plaques associated with neurodegenerative disorders such as Alzheimer’s disease [41,42].

The best-studied biofilm-associated amyloids are curli fibres in *E. coli* and *Salmonella enterica*, which also occur in other enterics [41,43,44]. In *E. coli*, curli fibres are co-regulated with pEtN-cellulose [45,46], with the two fibres tightly associating into a composite material and forming a large-scale matrix architecture within biofilms [24,29]. In many *E. coli* strains, curli fibres and cellulose are produced below 30 °C only, suggesting a major role in environmental biofilms. However, curli fibres and/or pEtN-cellulose can also be produced at 37 °C by certain commensal or pathogenic *E. coli* [47,48]. In the human intestine, curli fibres promote inflammation and can even trigger autoimmunity; i.e., also acting as a virulence factor [40,49,50,51]. Various *Pseudomonas* species produce Fap fibres (for functional amyloids in Pseudomonas) that have properties similar to curli fibres in enteric bacteria and that contribute to cellular aggregation in biofilms as well as to the virulence of pathogenic pseudomonads [52]. Also, Gram-positive bacteria use functional amyloids or amyloid-like fibres as biofilm matrix components. These include TasA fibres made by *Bacillus subtilis* which feature both alpha-helical and cross-beta sheet regions, with the fraction of the latter increasing at acidic pH [53,54]. *Staphylococci* generate various extracellular matrix-localized fibres from phenol-soluble modulins and other proteins, although these fibres do not seem to have all the properties of classical amyloids [39,40]. Finally, *Streptococcus mutans*, which plays an important role in the formation of dental plaque and the oral dysbiosis leading to caries [55], does not only produce exopolysaccharides it its biofilms, but also several exoproteins that can form amyloid fibres (summarized in [39]).

As bacterial biofilms are highly diverse in their structure and matrix composition, several model systems are currently used in research addressing both the formation and the inhibition of biofilms [29,56]: (i) submerged biofilms that form on the walls of microtiter dishes in which the biofilms’ overall mass can be easily quantified by staining with crystal violet or other dyes, (ii) macrocolonies growing for extended times at a semi-solid surface (agar)/air interface that are highly amenable to genetic approaches, and (iii) pellicles forming at static fluid/air interfaces [56]. In searching for and characterizing anti-biofilm agents, submerged biofilm models have been widely used, which due to the low cost and simplicity of the procedure can also be adapted to high-throughput screening. The more structured, CR-stainable and genetically analyzable macrocolonies as well as pellicles have become important more recently, especially in attempts to clarify the molecular mechanisms of actions of anti-biofilm agents.

## 3. EGCG Can Interfere with Bacterial Biofilm Formation

Green tea and its major constituent polyphenol EGCG, as well as many other plant-produced polyphenols, have long been discussed as being beneficial for human health. which is usually attributed to their general anti-oxidant and anti-inflammatory properties [19,57]. EGCG was also among one of the first polyphenols to have its effects tested on bacteria. While relatively high concentrations of EGCG were found to be toxic for various bacterial species (summarized by [58,59]), sub-inhibitory concentrations indeed often show anti-biofilm activity. Thus, a mixture of green tea catechins, most notably EGCG, was observed to reduce the adherence of *S. mutans* to surfaces, to somehow interfere with bacterial glucosyltransferases involved in biofilm formation and to be anti-cariogenic in animals and humans (summarized by [60,61]). EGCG was found to reduce submerged biofilm formation in microtiter dishes as well as swarming of *Burkholderia cepacia* [62]. Similar results for submerged biofilm formation were also obtained with *Staphylococci* [63], in particular with a series of ocular isolates of *S. aureus* and *S. epidermidis*. Here, EGCG also reduced CR staining of colonies, and scanning electron microscopy revealed strongly reduced matrix (‘slime’) production when *Staphylococci* were grown on corneal epithelial cells in vitro [64]. Since then, submerged biofilm formation under laboratory conditions was demonstrated to be impaired by EGCG for many more bacteria and in particular pathogenic species, including enterohemorrhagic *E. coli* (EHEC) [65]; *P. aeruginosa* [66]; *Porphyromonas gingivalis* (a member of the oral microbiota) [67,68]; *Stenotrophomonas maltophilia* isolated from the lung of a CF patient (here, biofilm formation was also reduced by EGCG in a mouse model) [69]; *Enterococcus faecalis* [70]; *Campylobacter jejuni* [71]; *Shewanella baltica* [72]; *Streptococcus pneumoniae* [73]; again, *S. mutans* (where EGCG effects on dental biofilms were also tested in vivo; i.e., in dogs); [74] and *Fusobacterium nucleatum*, another oral bacterium associated with periodontitis [75].

## 4. Mechanisms of Action of EGCG on Bacterial Biofilms

In general, the early reports on EGCG’s effects were based on simply comparing the mass of bacteria adhered to microtiter dishes in the presence and absence of EGCG and remained mostly descriptive with respect to how the compound affected these biofilms. However, a few of the early as well as the more recent studies have begun to address the question of the potential mechanisms of action of EGCG. In some bacteria, such as *P. aeruginosa*, where biofilm formation involves intercellular communication by quorum sensing, EGCG can somehow disturb signaling via autoinducer-1/LuxR-dependent or autoinducer-2-dependent quorum sensing systems [62,66,72]. In several bacterial species, EGCG can also attenuate the expression or activity of specific virulence factors, as observed for instance for Shiga toxin in enterohemorrhagic *E. coli* (EHEC) [65,76] or staphylococcal enterotoxin B [77]. In *P. gingivalis*, EGCG down-regulates flagella as well as factors involved in tissue destruction and heme acquisition [68]. In *E. faecalis*, the EGCG-mediated down-regulation of certain virulence genes was shown to involve the formation of hydroxyl radicals [70]. In *S. pneumoniae*, EGCG seems to target specifically the virulence factors pneumolysin and sortase A [73]. Evidence of EGCG-mediated membrane damage, iron chelation and reduced synthesis of hemolysin and hydrogensulfide was reported for *F. nucleatum* [75]. Not all of these activities are necessarily direct, although the diversity of effects suggests that EGCG may target a variety of cellular factors. It is also not clear which of these effects are related to the anti-biofilm action of EGCG, although virulence and biofilm genes are often co-regulated in complex manners. An exception is *S. mutans*, where the anti-biofilm effect of EGCG seems related to its ability to reduce the expression of the three genes (*gtfB*, *gtfC*, *gtfD*) that encode the glycosyltransferases that synthesize the biofilm-associated exopolysaccharides [78,79]. Although a number of target processes have thus been identified, the molecular mechanisms by which EGCG directly interferes with these remained mostly elusive in these studies.

In recent searches for molecular mechanisms targeted by EGCG, which are specifically related to biofilms, several strategies have been followed and combined: (i) theoretical considerations based on known molecular mechanisms of biofilm formation and control [12,80] to pinpoint appropriate targets for inhibition; (ii) the use of plate-grown macrocolony biofilms as an experimental biofilm system that—in contrast to microtiter dish-grown biofilms—allows to genetically characterize targets and mechanisms of action [23,24,81]; and (iii) taking into account reports on how EGCG acts in other systems, in particular in human biochemistry and physiology (see below), to get hints on direct molecular mechanisms of action that may also occur in bacteria.

Appropriate targets for the inhibition of biofilm formation should be components or processes that are crucial for the essential properties of most bacterial biofilms. While quorum sensing (QS) is not as generally involved in biofilm control as initially assumed, and the biochemical nature of QS signaling molecules is diverse, two other biochemical processes are widely conserved in biofilms: the formation of amyloid fibres in many biofilm matrices [36] and the regulation by the second messenger bis-(3′,5′)-cyclic diguanosine monophosphate (c-di-GMP), which rather ubiquitously activates exopolysaccharide synthases and/or the expression of numerous genes involved in bacterial biofilm formation [35,82,83,84,85]. Targeting a matrix component or process such as amyloidogenesis would have the additional advantage that an inhibitory molecule could act from outside; i.e., it would not have to traverse the entire cell envelope. This would be necessary to target for instance the cytoplasmic c-di-GMP-synthesizing diguanylate cyclases, which all share the same catalytic mechanism in their GGDEF domains (with this name standing for the highly conserved amino acids that define the active centre) [86]. With respect to activities in a human biochemical context, EGCG was shown to prevent amyloid formation in vitro by the A-beta and alpha-synuclein peptides, which are associated with Alzheimer’s and Parkinson’s diseases, respectively, by inducing the formation of non-toxic off-pathway oligomers [87,88,89]. Furthermore, EGCG inhibits amyloid fibre formation and even disrupts preformed fibres of a mutant version of transthyretin which is associated with familial amyloidotic polyneuropathy, where these extracellular fibres accumulate in connective tissue [90].

**Figure 1:**
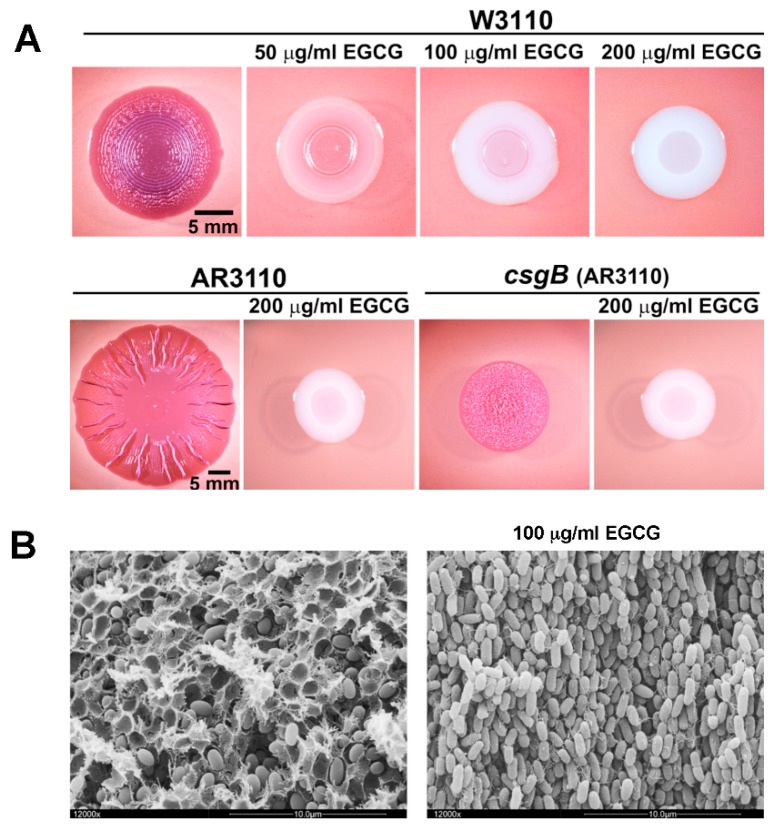
Epigallocatechin-3-gallate (EGCG) prevents the formation of amyloid curli fibres and pEtN-cellulose; i.e., the two major components of the extracellular matrix of macrocolony biofilms of *E. coli*. (**A**) Macrocolony biofilms of the standard *E. coli* K-12 strain W3110 (which produces only curli fibres), its derivative strain AR3110 (which produces curli fibres and pEtN-cellulose) and the *csgB* mutant derivative of strain AR3110 (which produces only pEtN-cellulose) were grown on salt-free complex medium agar plates for five days at 28 °C. Plates were supplemented with Congo red (which binds to and stains curli fibres as well as pEtN-cellulose fibrils) and the indicated concentrations of EGCG, which results in a white colour indicating an absence of the polymeric matrix components. Note that colony wrinkling patterns show characteristic differences depending on whether the matrix consists of the curli/pEtN-cellulose composite or of either component alone. (**B**) High-resolution scanning electron micrographs show tight surrounding of *E. coli* cells by the composite curli/pEtN–cellulose matrix inside a macrocolony biofilm of strain AR3100 (left panel), whereas no matrix is present with EGCG during growth (right panel). The few filamentous structures visible in the right panel are flagella, which are unaffected by EGCG. The photographs shown here were previously published [80] under the Creative Commons Attribution- NonCommercial-NoDerivatives license (CC-NC-ND); ^©^ the author.

**Figure 2:**
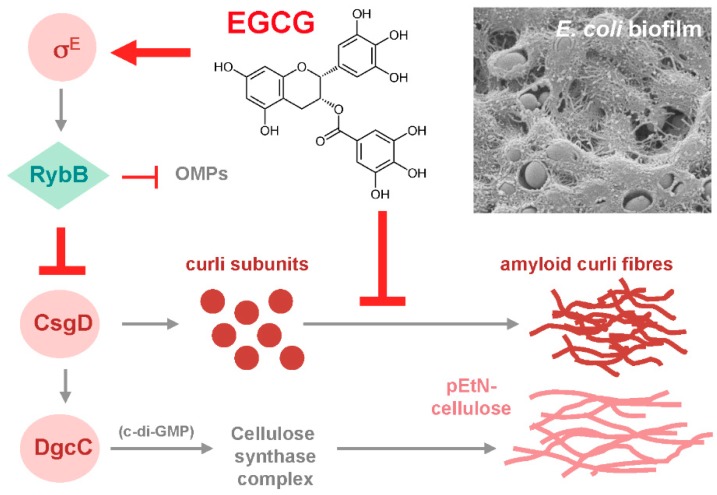
Summary of the mechanisms of action of EGCG on *E. coli* macrocolony biofilms. EGCG interferes with the production of both amyloid curli fibres and pEtN-cellulose fibril—which together form the fibrous matrix material visible in the scanning electron micrograph of the surface of an *E. coli* macrocolony biofilm shown in the upper right corner—by two separate but synergistically acting processes: (i) EGCG directly interferes with the formation of amyloid fibres from curli subunits; and (ii) EGCG induces the σ^E^ (RpoE)-mediated cell envelope stress response, which drives increased synthesis of the small regulatory RNA RybB, which in turn reduces the expression of CsgD as well as of many outer membrane proteins (OMPs). The biofilm regulator CsgD is required for the expression of the curli subunits as well as of DgcC, the diguanylate cyclase that produces the second messenger c-di-GMP required to activate cellulose synthase as well as the associated phosphoethanolamin (pEtN) modification system. The figure was previously published [80] under the CC-NC-ND license; ^©^ the author.

Since these considerations suggested biofilm amyloid formation as a prime candidate for being targeted by EGCG, the action of the compound was tested with macrocolony biofilms of commensal and pathogenic *E. coli* strains [80], where the wrinkled morphotype depends on the high production of amyloid curli fibres and pEtN-cellulose [24,29]. EGCG was indeed found to essentially eliminate the entire CR-stainable extracellular matrix; i.e., curli fibres as well as pEtN-cellulose (Figure 1). This drastic EGCG effect is due to a combination of (i) an anti-amyloidogenic activity, which results in curli subunits (or oligomers) remaining soluble in sodium dodecyl sulfate (SDS) and diffusing into the agar-containing medium below the biofilm, and (ii) triggering the cell surface stress response pathway governed by the alternative sigma factor RpoE (σ^E^; summarized in Figure 2). RpoE induces the small regulatory RNA RybB, which directly binds to and interferes with the translation of the mRNA for the biofilm regulator CsgD, which in turn is required to transcribe the curli genes as well as the gene for a diguanylate cyclase that is essential to specifically activate cellulose synthase [80]. How exactly EGCG triggers the RpoE-mediated cell envelope stress response is not yet clear; in part, because the signal input generally is the least understood part of this stress response. However, EGCG was observed to interact with lipid bilayers in cell membranes [91,92] and to induce small perforations and grooves in the cell surface of EHEC; i.e., to clearly induce cell envelope damage [93].

Besides showing anti-amyloidogenic activity of EGCG in a bacterial biofilm model, this study [80] thus also revealed a novel target for biofilm interference–cell envelope stress responses, which in multiple and complex manners respond to protein folding and assembly stress at the bacterial surface and/or cell envelope. Under such stress conditions, these responses generally tune down the additional synthesis of proteins destined for the cell envelope [94,95,96,97]. This includes proteins involved in large molecular ‘construction sites’ such as the trans-cell envelope machineries for the controlled synthesis of amyloid fibres and exopolysaccharides [80]. If this applies also to the subunits of conjugative pili, it may explain the observation that EGCG can reduce conjugative R plasmid transfer [98]. Without even having to enter bacterial cells, EGCG thus highly efficiently interferes with the macrocolony biofilm formation of *E. coli* by synergistically targeting two crucial processes.

In parallel, two studies with other bacterial amyloids also found these to be directly targeted by EGCG. This applies to Fap fibres in certain types of *P. aeruginosa* biofilms, where the major subunit FapC is remodeled into oligomers by EGCG, resulting in less stiff biofilms [99]. Since Fap fibres play an important role in *P. aeruginosa* biofilms in the infected CF lung, a nebulized administration of EGCG to CF patients was proposed as a potential treatment option which is worth further study [99]. The *fap* genes occur in various branches of proteobacteria, including a number of pathogens, and besides rendering biofilms highly aggregative and adherent, Fap fibres also seem to act as virulence factors [40]. Accordingly, the *fap* operon was highly activated in acute burn and chronical surgical wound infections of *P. aeruginosa* in a mouse model [100]. Another line of evidence shows that EGCG prevents fibril formation from the phenol-soluble modulins (PSM) of *Staphylococcus* species and can even disaggregate preformed fibrils [101]. PSMs have been shown to contribute to virulence in various ways, although it is still under debate whether these toxic activities require the fibrous forms of PSMs [40]. Given that *P. aeruginosa* and *S. aureus* are major biofilm-forming pathogens in chronic wound infection [102,103,104], the interference of EGCG with the biofilm-associated amyloid or amyloid-like fibres of these two pathogens and a putative therapeutic use of EGCG in this context certainly deserve further study.

## 5. Can EGCG Act Synergistically with Antibiotics on Bacterial Biofilms?

The high antibiotic tolerance of bacteria residing in biofilms severely impedes the antibiotic treatment of many chronic infections [5,6,8,105,106]. Therefore, drugs that can prevent biofilm formation are believed to also reduce biofilm-associated antibiotic tolerance, thereby allowing antibiotics to act more efficiently on biofilm-associated infections [11,12,17]. However, biofilm-related antibiotic tolerance is multifactorial, just as biofilm formation as a whole, is complex and involves many different molecular processes [107]. Certain extracellular matrix components can restrict diffusion and trap distinct antibiotics; i.e., the matrix can act as an effective filter that protects bacterial cells inside a biofilm against antibiotic exposure [108,109,110]. The viscoelastic behavior of submerged biofilms also affects water channels and thereby can influence rates of antibiotic penetration [111]. Moreover, most cells in a densely populated biofilm are in a physiological state of stationary phase, which renders cells not only tolerant against antibiotics that target the growing cell wall, but also induces pronounced multiple stress resistance which may involve changes in certain antibiotic target structures and processes [56,112,113,114]. Furthermore, distinct antibiotic efflux pumps were found to be specifically induced in biofilm-dwelling bacteria [115,116]. Finally, the formation of persister cells, which are in a non-growing and/or dormant state, contribute to antibiotic tolerance in biofilms [117].

Because of this complexity, a given anti-biofilm agent is unlikely to counteract antibiotic tolerance in general, just as it is unlikely to target all processes involved in biofilm formation; after all, it is now clear that biofilm formation is not a process governed by a single hierarchically organized developmental pathway [56,118,119,120]. As a consequence, a specific anti-biofilm agent such as EGCG may contribute to suppress tolerance against a specific antibiotic in biofilms of a specific bacterial species, but ‘magic bullet’ synergistic activities with various antibiotics against biofilms in general are not to be expected. On the other hand, distinct activities of EGCG, which affect different molecular targets in bacterial cells, may combine to counteract both the more classical antibiotic resistance as well as biofilm-associated antibiotic tolerance. Notably, the underlying molecular mechanisms (e.g., efflux pumps) are often principally similar, no matter whether these are acquired via mobile genetic elements or are part of the ‘normal’ physiological repertoire activated in biofilms as described above.

Nevertheless, synergy between EGCG and certain antibiotics has been found in a series of studies, although earlier studies in particular did not focus on counteracting biofilm-associated antibiotic tolerance specifically. Thus, synergy of EGCG with β-lactams was found with methicillin-resistant *S. aureus* (MRSA) [121,122,123]. By binding to peptidoglycan, EGCG restored antimicrobial activity of penicillin against a penicillinase-producing *S. aureus* [124]. Besides reducing biofilm formation by *P. gingivalis*, EGCG affected the growth of this oral bacterium synergistically with metronidazole [68]. EGCG also seems to interfere with the activities of certain antibiotic efflux pumps, as was shown for tetracycline efflux in *Staphylococci* [63] and ciprofloxacin efflux in β-lactamase-producing *Klebsiella pneumoniae* [125]. A biofilm-specific synergy was reported for *P. aeruginosa*, where EGCG remodels not only Fap fibres in the biofilm matrix as described above, but also increases the susceptibility of the biofilm to tobramycin [99].

On the other hand, specific anti-biofilm activity of EGCG and a reduction in biofilm-associated antibiotic tolerance are not necessarily coupled. Under certain conditions, EGCG was found to even promote biofilm formation and to increase tobramycin resistance of *P. aeruginosa* [126]. The underlying molecular mechanisms have not been clarified but it is conceivable that by reducing amyloid fibre formation, EGCG may lead to better exposure of some biofilm-promoting adhesin(s) on the bacterial surface. Similarly, exposure to EGCG reduced susceptibility to several antibiotics in *S. aureus* [127].

Overall, more studies of potential synergies between EGCG and distinct antibiotics specifically in biofilms of relevant pathogenic bacterial species are required while keeping in mind that even opposite and thus counterproductive effects can occur. Studies of synergies could be guided by the increasing knowledge about the direct molecular targets of EGCG. For instance, the finding that EGCG strongly reduces the formation of amyloid fibres and pEtN-cellulose fibrils [80,99,101] suggests putative synergy specifically with antibiotics that are tolerated by biofilms due to their binding by these matrix components.

## 6. Why do Plants Produce Antibiofilm Agents such as EGCG?

There is a huge body of literature on the therapeutic use of EGCG for various human diseases, including neurodegenerative diseases, cancer and infectious diseases [59,128,129,130]. However, EGCG-producing plants have neither evolved nor been bred to help humans treat diseases. So, why do plants produce catechins and epicatechins in sometimes strikingly high concentrations, as is the case for EGCG in *Camellia sinensis* [131]? Polyphenols in general are anti-oxidants and can protect the producing plants, for example, against UV irradiation [132]). They have also been implicated in resistance against bacterial or fungal plant pathogens [133,134,135], which is consistent with effects on the expression of certain virulence genes as mentioned above.

As shown by recent studies [80,99,101], EGCG exhibits strong anti-amyloidogenic function at much lower concentrations than antimicrobial effects (with *E. coli*, for instance, the formation of amyloid curli fibres is inhibited at <50 μg/mL, whereas the minimal inhibitory concentration or MIC is >10-fold higher)—a property indicating that EGCG is a potent anti-biofilm agent against bacterial biofilms that contain amyloid fibres as a major extracellular matrix component. The same applies to pEtN-cellulose fibrils, the production of which is equally inhibited by EGCG [80] and which are also common in bacterial biofilm matrices [30]. The general question why plants produce EGCG can thus be rephrased more specifically: do plants come into contact with bacterial biofilms containing amyloid fibres or pEtN-cellulose to the point that they have to antagonize their deposition or formation on their surfaces?

The life cycle of enteric bacteria such as *E. coli* or *Salmonella*, which occur in mammals, birds and other animals, includes shedding into the environment [136]. Due to their general and specific stress responses, these bacteria are well equipped to survive or even grow in very different and stressful environments outside their hosts [137]. This includes bacterial life on the leaves of plants, i.e., in the phyllosphere, where bacteria are exposed to nutrient limitation, desiccation and large changes in temperature and UV irradiation [138,139]. Notably, from the bacterial perspective, firmly attaching to plant leaves seems an attractive strategy to find a way back into the intestines of plant-eating mammalian hosts. In fact, a number of reports have shown that curli fibres and pEtN-cellulose are induced and involved in the formation of enteric biofilms on plant surfaces, both in the phyllosphere and the rhizosphere [140,141,142,143,144,145].

On the other hand, plants—as with all macroorganisms—have to generally control bacterial growth on their surfaces and, in particular, have to defend themselves against being colonized by phytopathogenic bacteria. To achieve this, plants have evolved both constitutive and inducible defense mechanisms based on the use of chemicals. While constitutive defense strategies include the production of essential oils and various other secondary metabolites, inducible defense uses specific recognition events that trigger molecular responses that lead to the production of reactive oxygen species, toxic phytoalexins and signaling compounds such as salicylic acid, jasmonic acid and ethylene [146,147,148,149]. Antimicrobial action as well as specific effects of these compounds on virulence mechanisms have been described in many studies [18,150]. However, instead of completely clearing bacteria and thereby opening new niches for potentially worse pathogens, the plant phylosphere and rhizosphere are associated with a complex microbiota [138,151]. Thus, plants actively promote the growth of beneficial bacteria [152] and interfere with the growth of specific pathogens; e.g., by targeting the latter’s specific virulence mechanisms [18].

Chemical communication implies that plants have to maintain their microbiota in an accessible and manageable state; i.e., plants should ‘domesticate’ their microflora by inhibiting or at least controlling bacterial biofilm formation. Hence, plants can be expected to have evolved an arsenal of effective anti-biofilm compounds. Seen from the plant perspective, producing high levels of EGCG most likely is a strategy to interfere with the formation of amyloid and/or pEtN-cellulose-containing bacterial biofilms on their surfaces. Directly targeting these extracellular matrix components also seems an attractive strategy because, with this mode of action, anti-biofilm agents do not have to penetrate bacterial cell envelopes, in contrast to compounds that would directly affect intracellular anti-biofilm targets such as c-di-GMP-synthesizing diguanylate cyclases. Similarly, signaling pathways that respond to cell envelope stress can be triggered from outside, which allows indirect manipulation of bacterial gene expression or intracellular signaling. Notably, EGCG—like many polyphenols, a water-soluble compound—acts from outside both on biofilm matrix fibre assembly and on a cell envelope stress response that down-regulates the biosynthesis of the matrix fibre building blocks (summarized in Figure 2). Finally, based on these general considerations as well as the specific findings for EGCG, we may expect additional plant secondary compounds to show anti-biofilm activity—a hypothesis that could direct future experimental studies.

## 7. Conclusions and Perspectives

As a compound with anti-amyloidogenic properties and the ability to interfere with the production of bacterial pEtN-cellulose fibrils, EGCG is a potent inhibitor of the formation of certain types of biofilms, namely those that use amyloid fibres and pEtN-cellulose as major extracellular matrix components. As described above, this has been studied in most detail with *E. coli* [80], but seems to apply for instance also to bacteria in oral biofilms [153]. Accordingly, EGCG alone or added to materials used in dentistry has yielded promising results with respect to reducing the growth and/or surface adhesion of *S. mutans* and caries formation [60,78,154,155,156,157,158]. Furthermore, EGCG is effective against the formation of amyloids or amyloid-like fibres in biofilms of *P. aeruginosa* and *S. aureus* [99,101]. The anti-biofilm activity of EGCG against these two bacterial species, which are commonly found in chronic wound infections, may thus add to other wound-healing effects of EGCG [159,160,161,162]. Topical applications against oral or wound biofilms would also be less affected by the limited stability and bioavailability of EGCG due to oxidation and degradation by the intestinal microbiota [163,164,165,166].

However, EGCG neither seems a general anti-biofilm agent nor do its specific anti-biofilm properties necessarily lead to a reduction in biofilm-associated antibiotic tolerance. Under certain conditions, EGCG was found to even promote biofilm formation and/or to increase tolerance of certain antibiotics [126,127]. Certain cell envelope stress signaling pathways triggered by EGCG could lead to a stress-protective response that actually promotes biofilm formation and the associated antibiotic tolerance. Given the complexities of bacterial physiological reactions, beneficial effects of EGCG against biofilms thus seem quite specific and conditional, with counterproductive effects not being excluded.

Amyloid curli fibres of enteric bacteria are not only important biofilm matrix components but can also elicit pro-inflammatory effects in human hosts [51]. The temperature regulation of curli is relaxed in many commensal or pathogenic intestinal *E. coli*; i.e., these strains express curli also at human body temperature [47,48]. Antibodies against CsgA, the major curli subunit, have been detected in serum from sepsis patients but not in healthy controls, and curli was shown to induce a variety of proinflammatory cytokines [167], with curli being recognized by Toll-like receptors 1 and 2 [49,168,169]. Curli–DNA composites which can form in biofilm matrices can also stimulate the auto-immune response underlying systemic lupus erythematosus [50]. Taken together, this suggests a detrimental role of amyloid fibres produced by members of the human gut microbiota, which may be relevant in the chronically inflamed intestine. Inflammatory bowel diseases are characterized by enteric blooms; i.e., a strong increase in the relative abundance of mostly *E. coli*. These bacteria benefit from and promote inflammation, thereby stabilizing the inflamed state [170]. That green tea polyphenols are beneficial in the treatment of inflammatory bowel disease has been attributed to their effects on various components of the host pro-inflammatory control network [170,171,172,173]. However, also processes on the bacterial side, i.e., a putative involvement of pro-inflammatory curli fibres in chronic intestinal inflammation as well as the inhibition of curli production by EGCG, certainly deserve further study.

In general, a mutual interplay between EGCG and the gut microbiota seems to be currently emerging as a new intriguing field of study [167]. EGCG has a beneficial effect in cancer therapy [165,174], and specific microbiota-produced metabolites derived from EGC and EGCG were shown to interfere with the proliferation of cervical cancer (HeLa) cells [175]. Both EGCG and its microbiota-generated derivatives can scavenge toxic cellular reactive metabolites involved in the development of various chronic diseases [176]. EGCG was also found to prevent or even counteract high-fat diet-induced changes in the gut microbiota [177,178,179]. The specific processes underlying these beneficial effects of EGCG are not fully understood, but it is already apparent that they are multifactorial. Direct anti-oxidative effects by scavenging toxic reactive metabolites and the prevention of inflammation—both by targeting specific components of the innate immune system and by inhibiting the production of pro-inflammatory bacterial amyloids—are likely to operate synergistically in the molecular mechanism of action of EGCG.

Humans have used plant-derived materials for medical purposes for a long time without knowing or even asking how and why these agents were effective. Molecular biology is now unraveling the molecular mechanisms of action. In addition, the ecophysiological relationships between plants, bacteria, animals and humans now reveal why plants have evolved anti-amyloidogenic compounds such as EGCG. These compounds do not only prevent the formation of biofilms on plant surfaces, but at the same time can combat biofilms in chronic infections and even seem of value against toxic amyloid-associated neuropathological diseases.
